# Measurement of time delay for a prospectively gated CT simulator

**DOI:** 10.4103/0971-6203.62196

**Published:** 2010

**Authors:** M. Goharian, R. F. H. Khan

**Affiliations:** 1 Department of Medical Physics, Tom Baker Cancer Center, 1331 29^th^ Street NW, Calgary, Alberta, Canada, T2N4N2; 2 Department of Oncology, Physics and Astronomy, University of Calgary, 1331 29^th^ Street NW, Calgary, Alberta, Canada, T2N4N2

**Keywords:** CT simulator, prospective gating, time delay

## Abstract

For the management of mobile tumors, respiratory gating is the ideal option, both during imaging and during therapy. The major advantage of respiratory gating during imaging is that it is possible to create a single artifact-free CT data-set during a selected phase of the patient's breathing cycle. The purpose of the present work is to present a simple technique to measure the time delay during acquisition of a prospectively gated CT. The time delay of a Philips Brilliance BigBore™ (Philips Medical Systems, Madison, WI) scanner attached to a Varian Real-Time Position Management™ (RPM) system (Varian Medical Systems, Palo Alto, CA) was measured. Two methods were used to measure the CT time delay: using a motion phantom and using a recorded data file from the RPM system. In the first technique, a rotating wheel phantom was altered by placing two plastic balls on its axis and rim, respectively. For a desired gate, the relative positions of the balls were measured from the acquired CT data and converted into corresponding phases. Phase difference was calculated between the measured phases and the desired phases. Using period of motion, the phase difference was converted into time delay. The Varian RPM system provides an external breathing signal; it also records transistor-transistor logic (TTL) ‘X-Ray ON’ status signal from the CT scanner in a text file. The TTL ‘X-Ray ON’ indicates the start of CT image acquisition. Thus, knowledge of the start time of CT acquisition, combined with the real-time phase and amplitude data from the external respiratory signal, provides time-stamping of all images in an axial CT scan. The TTL signal with time-stamp was used to calculate when (during the breathing cycle) a slice was recorded. Using the two approaches, the time delay between the prospective gating signal and CT simulator has been determined to be 367 ± 40 ms. The delay requires corrections both at image acquisition and while setting gates for the treatment delivery; otherwise the simulation and treatment may not be correlated with the patient's breathing.

## Introduction

In recent years, radiotherapy treatment planning has increasingly used conformal techniques; an advanced treatment technique allows physicians to target a tumor volume, while sparing normal tissues. In conformal techniques, a high level of geometric accuracy is necessary to ensure delivery of the prescribed dose to the target volume. Organ and tumor motion can cause both random and systematic errors.[1,2] There are different ways to compensate for tumor motion, for example, breath-holding techniques, respiratory gating, and tumor-tracking. The goal of these approaches is to generate an individualized target volume. The most common approach to compensate for respiration-induced motion is four-dimensional radiotherapy, where the respiratory motion is compensated for during imaging, planning, and treatment delivery.[3] An essential component of this technique is four-dimensional computed tomography (4DCT). In 4DCT, a patient is allowed to breathe freely while the respiratory trace is recorded using various monitoring devices. There are two different ways to use CT in gated mode: ‘prospective’ and ‘retrospective’ gating. In prospective gated imaging, the CT scanner acquires images only at one phase or at a limited portion of the respiratory cycle. The CT couch is then moved to the next indexed position. The process is repeated to obtain a single volumetric CT dataset at a specific phase of the respiratory cycle. Obviously, it relies on the fact that the relationship between the tumor motion and external markers is established *a priori* and the breathing amplitude and phase remain smooth throughout image acquisition. In the retrospective gated scanning (also called 4DCT), the CT scanner acquires images continuously during all phases at a higher pitch. Each collected image is identified by phase as well as the couch position. Data can then be sorted in several phase bins according to the breathing trace, resulting in 1–10 CT datasets.

**Figure 1:**
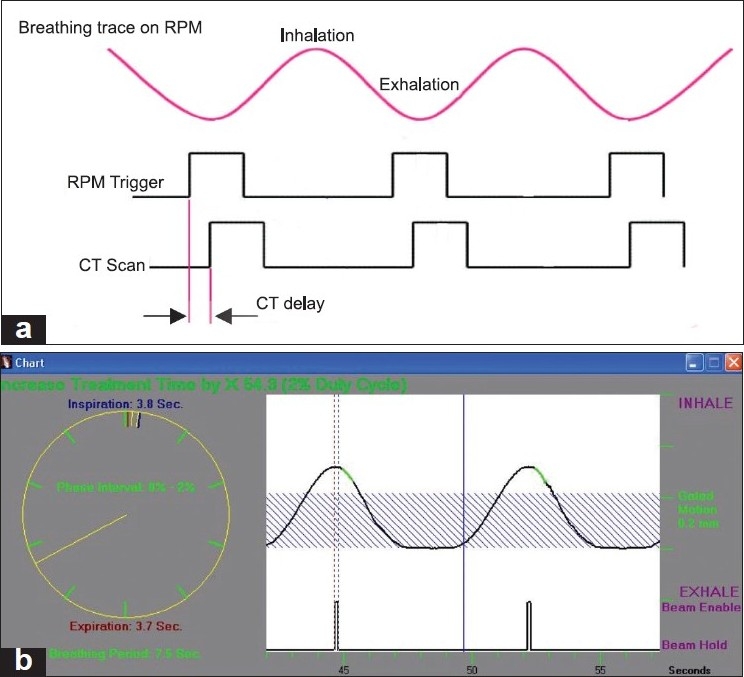
Time delay for prospectively gated CT acquisition. a) Time diagram shows a time lag between the RPM trigger pulse and the CT control pulse and b) the actual position of the CT scan is shown in green color

The prospective gated technique can be used to reduce motion-induced error in CT planning and treatment.[4–6] In prospective gating, the user is able to trigger an axial scan at a particular breathing level (amplitude mode) or phase [Figure 1a]. This is useful in patients who are not able to hold their breath during the scan, as it minimizes imaging artefacts due to respiratory motion. In this respect, a prospectively gated CT dataset may be useful for planning gated treatments. By matching the CT scan phase with the treatment phase it is assured that the treatment plan generated by the CT simulator delivers the desired dose to the tumor when it is in the right respiratory phase. If the CT simulation and the radiation treatment scan are not synchronized, a lower dose may be delivered to the tumor and the normal organs may be irradiated. Therefore, it is imperative to measure time delay in both the CT simulator and the linear accelerator.

Due to issues in establishing a correlation between the internal organ motion and the external markers, gated radiation therapy did not gain much acceptance. However, gated radiotherapy has shown success in target volumes where the organ motion and the external markers can be visually correlated, e.g., in breast tumors, where the planning target volume (PTV) includes the whole breast tissue. There is a scarcity of studies on time delay measurements for gated radiotherapy.[7,8] While several approaches have been published for establishing the correlation between internal organs motion and the external markers, it is important to study gating from the point of view of its impact on imaging. The purpose of the current work is to present an approach to measure the time delay of a CT simulator. In this study, a Philips Brilliance BigBore™ (Philips Medical Systems, Madison, WI) CT simulator was used for prospective gating. The respiratory motion signal generated from the RPM system is used to drive the CT scanner. The delay exists in the whole process of generation of control signal from respiratory signal including obtaining signal, processing the information and generation a control signal, and for the CT scanner to respond to the control signal. The overall delay from these steps is defined as the time delay of the gating system [Figure 1].

## Materials and Methods

### Scanning parameters

The RPM system used in this study consists of a marker block and an infrared camera that has been used in several studies.[9,10] The plastic block contains two infrared reflectors which are separated by 3 cm for the tracking purposes. An infrared camera is mounted at the CT table to track movement of the markers and thus produces the respiratory signal at the RPM computer terminal. Measurements were performed with the CT operated in prospective axial gating mode. The image acquisition settings for the Philips 16-slice CT scanner were as follows: 120 kVp, 140 mA, collimator setting of 16 × 1.5 mm, and 3-mm slice thickness. In gated acquisition, the signal from the scanner's injector port provides a triggering mechanism. The CT scanner waits for the signal from the RPM before starting the acquisition. Each signal received by the injector port will automatically trigger the CT for image acquisition. The trigger is in the form of a circuit closure and a simultaneous transistor–transistor–logic (TTL) output pulse commanded by the RPM system. The circuit closure and pulse are momentary and last for about 0.5 s, after which the circuit returns to an open position. Based on the time at which the RPM system triggers the scanner, the CT scan length, and the CT delay, the RPM system estimates the ‘X-ray ON’ interval. Each green segment of the respiratory waveform shown in Figure 1b indicates the estimated time during which a scan is being acquired. The portion of the waveform between the threshold and the beginning of the green segment represents the CT scan trigger delay time. After each trigger signal, the system cannot issue another one for a period of time equal to the triggering delay plus the scan time.

### Time delay measurement using motion phantom

**Figure 2:**
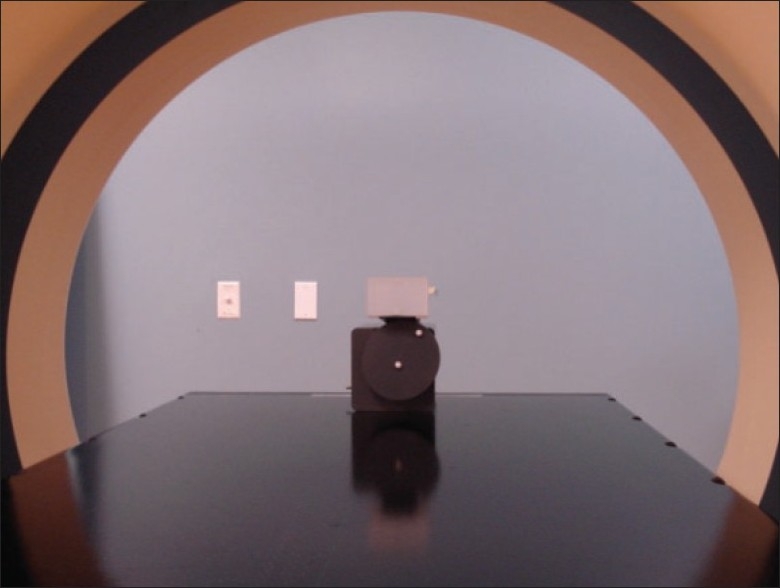
A modified motion phantom was used for time-delay measurement; the two plastic balls, on the axis and periphery, are visible

The motion phantom supplied by Varian has an elliptical wheel that rotates at approximately 9 revolutions per minute. The phantom was modified by placing two plastic ball bearings (BBs) on the rotating wheel: one BB placed along the axis of rotation and the other on the periphery, far from the axis of rotation of the wheel [Figure 2]. The relative positions of the images of the two BBs on a CT dataset were measured and then converted into phases. The topmost position of the off-center ball was identified to the end of inhale (zero phase) and the bottommost position to the end of exhale on the breathing trace. In this study, we acquired axial scans gated at ten different phases (0%, 10%, …, 90%) of the cyclic phantom signal with a 2% gating window. This corresponds to a time windowing of 146 ms for a total cycle time of 7.34 s. The width of the window was intentionally chosen as the minimum. The data were then processed using Matlab™ software (The Mathworks Inc., Natick, MA). The measured angular position of the off-center ball was converted into the phase. The measured phases were compared with the desired phases. The phase difference was converted to time delay by ascertaining the period of motion.

### Time delay determination using data file

The RPM gating system can generate a motion data file that can be processed to extract the respiration motion. The file is created in text format. It consists of a header and a data section. The header contains version, patient ID, data, and actual recording length. The data section of the file consists of amplitude, phase, time stamp, TTL-in, and TTL-out. The TTL ‘X-Ray ON’ status signal from the CT scanner was simultaneously recorded in a text file. A signal state transition from LOW (= 0) to HIGH (= 1) indicates the start of CT image acquisition. Knowledge of the start time combined with the phase and amplitude of real-time data from the external respiratory signal results in time-stamping of all images in the CT scan. The TTL-in and TTL-out status, in combination with the time information, was used to determine when (during the breathing cycle) a slice was acquired. The CT time delay was calculated as the difference between time stamps where both TTL-out and TTL-in were high (= 1) and where only TTL-in was high (= 1).

## Results

**Figure 3:**
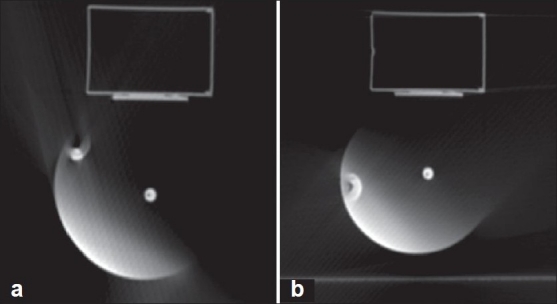
Axial CT images of the rotating wheel motion phantom; the images were acquired at a) 10% and b) 20% phases. The angular location of the off-center ball bearing (BB) in both images is different from the planned phases

**Figure 4:**
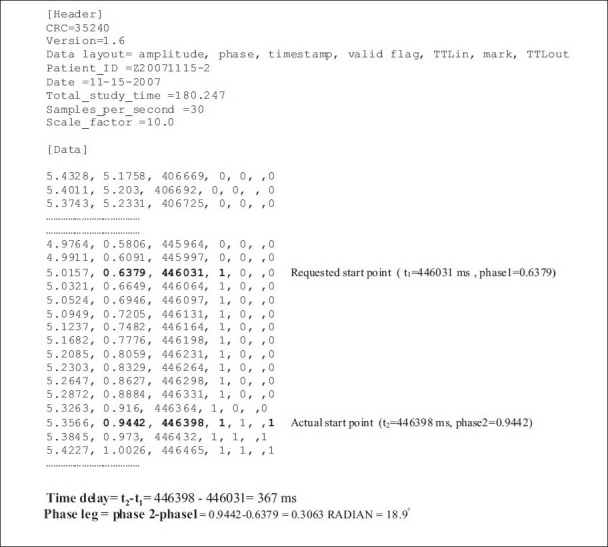
An extract of the RPM respiratory gating text file for determination of prospective gating delay

The time-delay information calculated from the motion file was verified experimentally with measurement. Both methods resulted in the same outcome. Figure 3 shows the position of the ball for a phase window at 20%, i.e., 20–22% (72°–79.2°) and at 10%, i.e., 10–12% (36°–43.2°). In the CT image, the ball appears at 98.1° instead of between 72°–79.2°. An excerpt of the text file is given in Figure 4.[11] Data in Table 1 show both the measured and actual phases using images of the moving phantom. The results show that for each phase bin there is, on average, a phase lag of 19° ± 2° between the calculated and the measured phase. This gives an estimate of the overall time delay of 367 ± 40 ms.

**Table 1:** Measured and actual angles for prospective gated CT

*Gate width %*	*Expected angle*	*Measured angle (±2°)*	*Difference*
0–2	0°–7°	25°	18°–25°
10–12	36°–43°	60°	17°–24°
20–22	72°–80°	98°	18°–26°
30–32	108°–115°	137°	22°–29°
40–42	144°–151°	173°	22°–29°
50–52	180°–187°	208°	21°–28°
60–62	216°–223°	243°	20°–27°
70–72	252°–259°	278°	19°–26°
80–82	288°–295°	311°	16°–23°
90–92	324°–331°	348°	17°–24°

Average: 19°-26°

## Discussion and Conclusions

In respiratory-gated imaging, the time delay in image acquisition should be known. The current version of the RPM software allows the user to compensate for the CT time delay as an input parameter in the CT interface for prospective axial gating. To this effect, most of the vendors require measurement or verification of time delay during the commissioning of CT simulator equipped with an external gating option. In this study, we present a straightforward approach to the measurement of the time delay of a CT simulator (a Philips scanner triggered by a Varian RPM system). The proposed technique was tested and found to be reliable as well as accurate. This approach can also be extended to determine time delay of other x-ray imaging systems such as electronic portal imaging devices (EPIDs) in ciné mode and flat-panel on-board imagers with a slight modification of a moving phantom. The time-delay effect requires correction at both image acquisition and treatment delivery stages. If the time delay during imaging and delivery are different, the simulation and treatment would not be correctly correlated in time.

A similar modified phantom was used by Starkschall *et al.*[12] to evaluate the accuracy of phase-binning for a retrospectively gated CT (4DCT) dataset. Using two plastic BBs instead of metal ball bearings (which were used by others), the high-density metal artefacts produced in images can be eliminated. We demonstrated that a commercially available motion phantom, with a little modification, can be used to determine the time delay for a CT simulator. The approach described by us is very easy to implement, as compared to others[8] that have been used for a single-slice CT scanner. This technique requires little setup time and the results can be verified with the help of the recorded data file.
